# Risk Factors for Cardiovascular Disease and Their Clustering among Adults in Jilin (China)

**DOI:** 10.3390/ijerph13010070

**Published:** 2015-12-23

**Authors:** Jianxing Yu, Yonghui Ma, Sen Yang, Kai Pang, Yaqin Yu, Yuchun Tao, Lina Jin

**Affiliations:** Epidemiology and Statistics, School of Public Health, Jilin University, Changchun, Jilin 130021, China; yjxjlu@163.com (J.Y.); mayh@jlu.edu.cn (Y.M.); yangs@jlu.edu.cn (S.Y.); pangk@jlu.edu.cn (K.P.); yuyq@jlu.edu.cn (Y.Y.)

**Keywords:** cardiovascular diseases, risk factors, clustering, prevalence

## Abstract

*Background*: Clustering of cardiovascular disease (CVD) risk factors constitutes a major public health challenge. Although a number of researchers have investigated the CVD risk factor clusters in China, little is known about the related prevalence and clustering associated with demographics in Jilin Province in China; this study aims to reveal that relationship. *Methods*: A cross-sectional survey based on a sample of 16,834 adults aged 18 to 79 years was conducted in Jilin in 2012. The prevalence and clustering of CVD risk factors were analysed through complex weighted computation. Quantitative variables were compared by the *t* test, and categorical variables were compared by the Rao-Scott-χ^2^ test. Finally, multivariable logistic regression analysis was used to evaluate the CVD risk factor clusters associated with demographics. *Results*: The prevalences of hypertension, diabetes, dyslipidemia, overweight and smoking were 37.3%, 8.2%, 36.8%, 47.3%, and 31.0%, respectively, and these risk factors were associated with gender, education level, age, occupation and family income (*p* < 0.05). Overall, compared with females, the adjusted ORs of ≥1, ≥2 and ≥3 risk factors clusters in males were 3.70 (95%CI 3.26 to 4.20), 4.66 (95%CI 4.09 to 5.31), and 5.76 (95%CI 5.01 to 6.63), respectively. In particular, the adjusted ORs of ≥1, ≥2 and ≥3 risk factors increased with age. *Conclusions*: CVD risk factor clusters are common among adults in northeast China, and they constitute a major public health challenge. More effective attention and interventions should be directed toward the elderly and toward persons with lower incomes and low levels of education.

## 1. Introduction

Cardiovascular disease (CVD) is not only a serious threat to human health, but also an important contributor to the total costs of medical care worldwide [1]. According to the statistics, 30% of people die from CVD [2]. As a major cause of mortality, CVD is increasing at an alarming rate in China [3], and the related economic burden from 2005 to 2015 was estimated to be $550 billion [4].

Hypertension, diabetes, dyslipidemia, overweight and smoking are five well-established major risk factors for CVD [5,6,7]. Furthermore, a number of studies have indicated that the prevalence of CVD risk factors has increased in China in recent decades [8,9,10,11]. It is also well-recognized in the literature that a combination of these risk factors in one individual increases the risk of CVD. Meanwhile, studies have also shown that CVD risk factors tend to cluster and that the risk for CVD increases substantially with each additional risk factor [12,13,14].

Numerous researchers have investigated CVD risk factor clusters in China, such as CVD risk factor clustering among ethnic groups by Li *et al.* [15] and the prevalence of CVD risk factor clustering among the adult population by Gu *et al* [16]. However, little is known about the prevalence and clustering of CVD risk factors associated with the demographics in Jilin Province, China. 

Jilin is located in the central part of northeast China (latitude 40°~46°, longitude 121°~131°), with approximately 27 million people and an annual average temperature of 4.8 °C. The prevalence of chronic disease in Jilin Province was measured by Jilin University and the Jilin Department of Health in 2012. Here we use that investigative data to identify the prevalence of CVD risk factor clusters associated with demographics, which is important for any future attempts to reduce the prevalence of CVD. Additionally, we reveal that cold climates may place people at risk of developing CVD risk factors through poor diets and restricted physical activity.

## 2. Materials and Methods 

### 2.1. Study Population

We implemented a large-scale cross-sectional survey in Jilin Province in 2012, with a total sample size of 23,050. Multistage stratified random cluster sampling was used to select people aged 18 to 79 years old [17]. For the purpose of the present analyses, some subjects were excluded due to missing values (see the details in Parts 1 and 2 of the online Supplementary Material). Finally, a total of 16,834 people were included in the present analyses.

### 2.2. Ethics Statement

The ethics committee of the School of Public Health, Jilin University approved the study, and written informed consent was obtained from all of the participants before data collection. 

### 2.3. Data Collection and Measurement

All data were collected by direct interviews to ensure uniformity and accuracy. The questionnaire included demographics (e.g., gender, age, education, *etc.*), health-related behaviors (e.g., smoking, drinking, *etc.*) and anthropometric measurements (e.g., height, weight, hypertension, diabetes, *etc.*). Before the interviews, the investigators confirmed the identities of each participant. In addition, a second check to the survey was set that day to ascertain the validity of each question [17] (see the details in Parts 4 and 5 of the online Supplementary Material).

Height and weight were measured according to a standardized protocol and techniques, with the participants wearing clothing but no shoes. Blood pressure was measured using a mercury sphygmomanometer by trained professionals, and the subjects rested for at least 5 min before being measured [18]. After an overnight fast, fasting blood glucose and serum lipids were measured before breakfast using a Bai Ankang fingertip blood glucose monitor (Bayer, Leverkusen, Germany) and a MODULE P800 biochemical analysis machine (Roche Co., Ltd., Shanghai, China), respectively [19] (see the details in Part 6 of the online Supplementary Material).

### 2.4. Assessment Criteria

A CVD risk factor cluster was defined as at least two major factors clustered in one individual, and the five major risk factors were defined clearly as follows [20]: dyslipidemia was defined as using lipid-lowering drugs or having one or more of the following: triglyceride (TG) ≥ 1.7 mmol/L, total cholesterol (TC) ≥ 5.2 mmol/L, high-density lipoprotein cholesterol (HDL-C) < 1.0 mmol/L and low-density lipoprotein cholesterol (LDL-C) ≥ 3.4 mmol/L [21]. Hypertension was defined as resting systolic blood pressure (SBP) ≥140 mmHg and/or diastolic blood pressure (DBP) ≥ 90 mmHg and/or the use of antihypertensive medication in the past two weeks [22]. Diabetes was defined as the use of hypoglycemic agents or a self-reported history of diabetes or fasting blood glucose (FBG) of 7.0 mmol/L or more [23]. Overweight was defined as a body mass index (BMI) ≥ 24 kg/m^2^ [24]. Smoking was defined as having smoked at least one cigarette per day over the past 30 days [25]. 

### 2.5. Statistical Analyses

The estimations and comparisons of prevalence were weighted to the standard population in the 6th national general investigation in Jilin Province. The weights (in complex weighted computation) were used to adjust for differing response proportions, selection probabilities and deviations in the sample compared with the standard population, particularly in terms of gender and age composition [26].

The data are presented as means ± standard deviations (SD) unless otherwise stated. In addition, quantitative variables were compared using the *t* test, and categorical variables were compared using the Rao-Scott-χ^2^ test. Finally, the CVD risk factor clusters were analyzed through logistic regression. All statistical analyses were performed using the complex samples function of IBM SPSS 20.0. (SPSS Inc., New York, NY, USA) Statistical significance was set at *p* < 0.05.

## 3. Results

Table 1 shows that BMI, SBP, DBP, TG, TC, FBG were all significantly higher in males than in females (*p* < 0.05). However, HDL-C was significantly higher in females than in males (*p* < 0.05). Meanwhile, age and LDL-C did not differ significantly by gender (*p* > 0.05). 

**Table 1:** Descriptive characteristics of participants by gender for CVD.

Variable	All (*n* = 16834)	Female (*n* = 9104)	Male (*n* = 7730)	*t*	*p* value
Age (year)	42.67 ± 14.49	43.03 ± 14.55	42.33 ± 14.43	1.864	0.062
BMI (kg/m^2^)	24.04 ± 3.81	23.74 ± 3.80	24.32 ± 3.81	−6.184	<0.001
SBP (mmHg)	128.49 ± 20.18	124.59 ± 21.23	132.17 ± 18.41	−18.976	<0.001
DBP (mmHg)	78.72 ± 11.58	76.4 ± 11.28	80.91 ± 11.44	−17.151	<0.001
TG (mmol/L)	1.88 ± 1.82	1.61 ± 1.39	2.13 ± 2.13	−14.406	<0.001
TC (mmol/L)	4.76 ± 1.07	4.72 ± 1.08	4.79 ± 1.05	−2.688	<0.001
LDL-C (mmol/L)	2.83 ± 0.87	2.82 ± 0.89	2.84 ± 0.86	−0.797	0.426
HDL-C (mmol/L)	1.37 ± 0.38	1.43 ± 0.36	1.32 ± 0.38	13.105	<0.001
FBG (mmol/L)	5.39 ± 1.66	5.27 ± 1.63	5.53 ± 1.69	−10.114	<0.001

BMI: body mass index, SBP: systolic blood pressure, DBP: diastolic blood pressure, TC: total cholesterol, TG: triglyceride, LDL-C: low-density lipoprotein cholesterol, HDL-C: high-density lipoprotein cholesterol, FBG: fasting blood glucose.

As shown in Table 2, the prevalences of the five risk factors differed significantly by gender. In addition, the prevalences were higher in males than in females (*p* < 0.01), especially for smoking. However, the prevalences did not differ significantly by residence (*p* > 0.05). Further, the prevalences of hypertension and diabetes increased with age (*p* < 0.001), but dyslipidemia, overweight and smoking first increased, and then decreased with age (*p* <0.05); the peak prevalences for each appeared in the age groups 55–64, 55–64, and 45–54, respectively. Except for diabetes and dyslipidemia, the prevalences of other risk factors showed decreasing trends with education level (*p* < 0.05), and the prevalences of hypertension, diabetes and smoking were significantly different by family income (*p* < 0.05); the prevalences of hypertension and diabetes decreased when family income increased (*p* < 0.05). Moreover, the prevalences of all of the risk factors were greater in manual than in mental labor (*p* < 0.001).

The subjects were divided into four groups according to the number of CVD risk factors (see details in Table 3). The prevalences of 2 ≥3 CVD risk factors had an increasing trend with age but a decreasing trend with education level. Concurrently, there was no obvious trends with family income or occupation. Except for residence, the number of CVD risk factors differed significantly by gender, age group, education level, family income and occupation.

Table 4 presents that the prevalences of ≥1, ≥2 and ≥3 CVD risk factors (RFs) increased with age but decreased with education level and family income. Moreover, there was no obvious trend with occupation. Finally, compared with the group of RFs = 0, the number of CVD RFs ≥ 1, RFs ≥ 2 and RFs ≥ 3 differed significantly by gender, age group, education level, family income and occupation (*p* < 0.001).

**Table 2:** Prevalences of CVD risk factors by demographic characteristics.

Category	Subcategory	Hypertension % (95%CI)	Diabetes % (95%CI)	Dyslipidemia % (95%CI)	Overweight % (95%CI)	Smoking % (95%CI)
Risk Factor	—	31.0 (30.1, 31.9)	8.2 (7.8, 8.7)	36.8 (35.8, 37.8)	47.3 (46.3, 48.4)	31.0 (30.0, 32.0)
Gender	Female	26.7 (25.6, 27.9)	7.4 (6.8, 8.0)	30.4 (29.1, 31.7)	43.6 (42.1, 45.1)	9.1 (8.4, 9.9)
	Male	35.0 (33.7, 36.4)	9.0 (8.3, 9.8)	42.9 (41.4, 44.3)	50.8 (49.3, 52.4)	51.6 (50.1, 53.1)
	*p* value	<0.001	0.001	<0.001	<0.001	<0.001
Residence	Rural	31.5 (30.2, 32.9)	8.3 (7.6, 9.0)	36.6 (35.1, 38.2)	46.4 (44.7, 48.0)	32.0 (30.5, 33.5)
	Town	30.6 (29.4, 31.8)	8.2 (7.5, 8.8)	37.0 (35.7, 38.3)	48.1 (46.7, 49.5)	30.2 (28.9, 31.5)
	*p* value	0.324	0.801	0.714	0.112	0.075
Age	18–	7.8 (5.5, 11.0)	0.6 (0.2, 1.5)	16.9 (13.6, 20.8)	21.2 (17.6, 25.4)	27.2 (23.3, 31.5)
	25–	12.7 (10.8, 14.7)	2.7 (1.8, 4.0)	30.6 (28.1, 33.2)	44.7 (42.0, 47.4)	31.6 (29.1, 34.2)
	35–	25.5 (23.9, 27.1)	5.3 (4.5, 6.3)	37.2 (35.4, 39.0)	50.0 (48.2, 51.8)	32.3 (30.6, 34.0)
	45–	41.6 (40.1, 43.1)	11.5 (10.6, 12.6)	44.4(42.8, 45.9)	56.1 (54.6, 57.6)	33.9 (32.4, 35.4)
	55–	53.5 (51.7, 55.3)	17.2 (15.9, 18.6)	48.4(46.6, 50.2)	56.6 (54.8, 58.3)	30.3 (28.7, 32.0)
	65–79	64.3 (61.3, 67.2)	18.6 (16.6, 20.8)	45.4(42.5, 48.3)	52.0 (49.0, 55.0)	25.9 (23.0, 29.0)
	*p* value	<0.001	<0.001	<0.001	<0.001	0.001
Education	Junior school	40.3 (38.6, 42.0)	12.6 (11.6, 13.7)	38.5 (36.7, 40.3)	49.6 (47.8, 51.5)	32.8 (31.0, 34.7)
	Junior high school	31.7 (30.1, 33.5)	7.4 (6.7, 8.3)	36.7 (34.8, 38.6)	47.5 (45.5, 49.5)	32.3 (30.4, 34.1)
	High school	29.5 (27.8, 31.3)	7.9 (7.0, 8.9)	38.6 (36.5, 40.7)	47.2 (44.9, 49.4)	31.9 (29.9, 34.0)
	Undergraduate	21.0 (19.0, 23.2)	4.7 (3.9, 5.6)	32.7 (30.4, 35.1)	44.7 (42.2, 47.3)	25.5 (23.4, 27.7)
	*p* value	<0.001	<0.001	<0.001	0.039	<0.001
Family income (Chinese Yuan)	<500	38.7 (36.7, 40.7)	11.2 (10, 12.4)	38.5 (36.5, 40.6)	50.1 (47.9, 52.3)	31.4 (29.5, 33.4)
	500–	34.6 (32.6, 36.8)	9.5 (8.4, 10.7)	38.2 (36.0, 40.5)	47.6 (45.3, 49.9)	29.3 (27.3, 31.4)
	1000–	30.4 (28.8, 32.1)	8.1 (7.3, 9.0)	36.3 (34.5, 38.2)	46.7 (44.7, 48.7)	30.0 (28.2, 31.8)
	2000–	27.4 (25.3, 29.5)	6.2 (5.4, 7.3)	37.1 (34.8, 39.6)	49.2 (46.6, 51.8)	33.7 (31.4, 36.2)
	3000–	25.9 (23.0, 29.0)	5.8 (4.5, 7.4)	33.7 (30.3, 37.2)	46.0 (42.2, 49.9)	32.7 (28.9, 36.7)
	*p* value	<0.001	<0.001	0.147	0.277	0.035
Occupation	Manual labor	29.3 (28.2, 30.4)	7.1 (6.5, 7.7)	35.2 (34.0, 36.5)	46.9 (45.5, 48.3)	37.2 (35.9, 38.5)
	Mental labor	24.0 (22.1, 26.1)	5.9 (5.1, 6.9)	33.7 (31.5, 36.0)	44.5 (42.0, 47.0)	26.8 (24.7, 29.1)
	Other *	41.5 (39.3, 43.7)	13 (11.8, 14.2)	43.5 (41.2, 45.8)	51.1 (48.7, 53.4)	20.2 (18.4, 22.1)
	*p* value	<0.001	<0.001	<0.001	<0.001	<0.001

*p* values were calculated with the Rao-Scott-χ^2^ test; * “other” included unemployed and retired people.

**Table 3:** Prevalences with the 5 CVD risk factors.

Category	Subcategory	The Number of CVD Risk Factors				χ^2^	*p* value
		0 (*n* = 3320)	1 (*n* = 4697)	2 (*n* = 4356)	≥3 (*n* = 4466)		
Gender	Female	36.1 (34.5, 37.8)	29.1 (27.7, 30.6)	19.8 (18.8, 20.9)	14.9 (14.1, 15.8)	1517.202	<0.001
	Male	13.3 (12.1, 14.5)	27.3 (25.9, 28.7)	27.9 (26.5, 29.2)	31.6 (30.3, 32.9)		
Residence	Rural	23.4 (21.6, 25.3)	28.7 (27.2, 30.2)	25.1 (23.8, 26.5)	22.8 (21.7, 24.0)	16.897	0.054
	Town	25.2 (23.9, 26.5)	27.8 (26.5, 29.1)	23.0 (21.9, 24.2)	24.0 (22.9, 25.2)		
Age	18–	50.1 (44.9, 55.2)	31.0 (26.6, 35.8)	14.7 (11.6, 18.4)	4.2 (3.1, 5.8)	2303.899	<0.001
	25–	33.9 (31.5, 36.4)	29.3 (26.9, 31.8)	21.1 (18.9, 23.5)	15.6 (13.6, 17.9)		
	35–	25.3 (23.8, 26.8)	29.7 (28.1, 31.3)	22.4 (20.9, 23.9)	22.7 (21.2, 24.3)		
	45–	13.8 (12.8, 14.9)	27.9 (26.6, 29.3)	26.7 (25.4, 28.1)	31.6 (30.1, 33.1)		
	55–	9.7 (8.7, 10.9)	23.6 (22.2, 25.2)	30.5 (28.9, 32.2)	36.1 (34.4, 37.8)		
	65–79	7.4 (6.2, 8.9)	25.1 (22.4, 28.0)	32.4 (29.8, 35.1)	35.1 (32.2, 38.1)		
Education	Junior school	15.6 (14.1, 17.2)	30.2 (28.5, 31.9)	27.5 (25.9, 29.1)	26.7 (25.2, 28.3)	364.427	<0.001
	Junior high school	23.5 (21.6, 25.4)	28.8 (26.9, 30.8)	24.3 (22.8, 25.9)	23.4 (21.9, 24.9)		
	High school	25.9 (23.5, 28.5)	25.8 (24.1, 27.6)	23.4 (21.7, 25.2)	24.8 (23.2, 26.6)		
	Undergraduate	33.7 (31.2, 36.2)	28.1 (25.8, 30.6)	20.1 (18.1, 22.1)	18.1 (16.4, 20.0)		
Family Income (Chinese Yuan)	<500	18.0 (15.9, 20.3)	29.4 (27.5, 31.4)	25.9 (24.2, 27.7)	26.7 (25.0, 28.5)	219.416	<0.001
	500–	22.5 (20.3, 24.9)	27.8 (25.8, 30.0)	25.4 (23.5, 27.4)	24.3 (22.5, 26.2)		
	1000–	25.5 (23.5, 27.6)	28.4 (26.7, 30.3)	22.7 (21.2, 24.3)	23.3 (21.9, 24.8)		
	2000–	24.6 (22.4, 26.9)	28.1 (25.8, 30.5)	24.3 (22.1, 26.5)	23.1 (21.2, 25.1)		
	3000–	28.5 (24.9, 32.5)	26.6 (23.2, 30.2)	23.8 (20.7, 27.2)	21.1 (18.3, 24.2)		
Occupation	Manual labor	22.8 (21.5, 24.1)	29.1 (27.9, 30.3)	25.1 (23.9, 26.3)	23.0 (22.0, 24.1)	90.579	<0.001
	Mental labor	32.0 (29.3, 34.8)	28.5 (26.2, 30.9)	19.6 (17.9, 21.5)	19.9 (18.3, 21.7)		
	Other *	20.9 (18.7, 23.3)	25.7 (23.6, 28.0)	25.4 (23.6, 27.2)	28.0 (26.2, 29.9)		

* “other” included unemployed and retired people.

**Table 4:** Prevalences with different numbers of CVD risk factors.

Category	Subcategory	The Number of CVD Risk Factors			
		0	≥1	≥2	≥3
Gender	Female	36.1 (34.5, 37.8)	63.9 (62.2, 65.5)	34.7 (33.4, 36.1)	14.9 (14.1, 15.8)
	Male	13.3 (12.1, 14.5)	86.7 (85.5, 87.9)	59.5 (57.9, 61.0)	31.6 (30.3, 32.9)
	*p* value	—	<0.001	<0.001	<0.001
Residence	Rural	23.4 (21.6, 25.3)	76.6 (74.7, 78.4)	48.0 (46.3, 49.6)	22.8 (21.7, 24.0)
	Town	25.2 (23.9, 26.5)	74.8 (73.5, 76.1)	47.1 (45.7, 48.5)	24.0 (22.9, 25.2)
	*p* value	—	0.123	0.226	0.041
Age	18–	50.1 (44.9, 55.2)	49.9 (44.8, 55.1)	18.9 (15.6, 22.8)	4.2 (3.1, 5.8)
	25–	33.9 (31.5, 36.4)	66.1 (63.6, 68.5)	36.7 (34.1, 39.5)	15.6 (13.6, 17.9)
	35–	25.3 (23.8, 26.8)	74.7 (73.2, 76.2)	45.1 (43.3, 46.9)	22.7 (21.2, 24.3)
	45–	13.8 (12.8, 14.9)	86.2 (85.1, 87.2)	58.3 (56.8, 59.8)	31.6 (30.1, 33.1)
	55–	9.7 (8.7, 10.9)	90.3 (89.1, 91.3)	66.6 (64.9, 68.3)	36.1 (34.4, 37.8)
	65–79	7.4 (6.2, 8.9)	92.6 (91.1, 93.8)	67.5 (64.5, 70.3)	35.1 (32.2, 38.1)
	*p* value	—	<0.001	<0.001	<0.001
Education	Junior school	15.6 (14.1, 17.2)	84.4 (82.8, 85.9)	54.2 (52.3, 56.0)	26.7 (25.2 ,28.3)
	Junior high school	23.5 (21.6, 25.4)	76.5 (74.6, 78.4)	47.7 (45.7, 49.7)	23.4 (21.9, 24.9)
	High school	25.9 (23.5, 28.5)	74.1 (71.5, 76.5)	48.2 (46.0, 50.5)	24.8 (23.2, 26.6)
	Undergraduate	33.7 (31.2, 36.2)	66.3 (63.8, 68.8)	38.2 (35.8, 40.7)	18.1 (16.4, 20.0)
	*p* value	—	<0.001	<0.001	<0.001
Family Income (Chinese Yuan)	<500	18.0 (15.9, 20.3)	82.0 (79.7, 84.1)	52.6 (50.3, 54.8)	26.7 (25.0, 28.5)
	500–	22.5 (20.3, 24.9)	77.5 (75.1, 79.7)	49.7 (47.3, 52.0)	24.3 (22.5, 26.2)
	1000–	25.5 (23.5, 27.6)	74.5 (72.4, 76.5)	46.1 (44.1, 48.0)	23.3 (21.9, 24.8)
	2000–	24.6 (22.4, 26.9)	75.4 (73.1, 77.6)	47.3 (44.8, 49.9)	23.1 (21.2, 25.1)
	3000–	28.5 (24.9, 32.5)	71.5 (67.5, 75.1)	44.9 (41.1, 48.8)	21.1 (18.3, 24.2)
	5000–	26.6 (19.3, 35.5)	73.4 (64.5, 80.7)	45.3 (37.9, 52.9)	22.5 (17.0, 29.2)
	*p* value	—	<0.001	<0.001	<0.001
Occupation	Manual labor	22.8 (21.5, 24.1)	77.2 (75.9, 78.5)	48.1 (46.7, 49.5)	23.0 (22.0,24.1)
	Mental labor	32.0 (29.3, 34.8)	68.0 (65.2, 70.7)	39.5 (37.2, 41.9)	19.9 (18.3,21.7)
	Other *	20.9 (18.7, 23.3)	79.1 (76.7, 81.3)	53.4 (51.0, 55.8)	28.0 (26.2,29.9)
	*p* value	—	<0.001	<0.001	<0.001

*p* values were obtained by constructing 2 × 2 contingency tables for other groups (RFs = 1, RFs = 2, RFs ≥ 3) compared with RFs = 0; * “other” included unemployed and retired people.

Table 5 shows that the males were more likely to have ≥1, ≥2 and ≥3 CVD risk factors than were females (*p* < 0.05). The adjusted ORs of RFs ≥ 1, RFs ≥ 2 and RFs ≥ 3 (*versus* RFs = 0) increased progressively with age. On the contrary, the adjusted ORs of RFs ≥ 1, RFs≥ 2 and RFs ≥ 3 (*versus* RFs = 0) decreased progressively with education level. Concurrently, there was no obvious trend with occupation or family income. 

**Table 5:** The logistic analysis of the CVD risk factor clustering among participants.

Category	Subcategory	The CVD Risk Factors and Adjusted OR (95%CI)		
		≥1	≥2	≥3
Gender	Female	1.00	1.00	1.00
	Male	3.70 (3.26,4.20)	4.66 (4.09, 5.31)	5.76 (5.01, 6.63)
Age	18–	1.00	1.00	1.00
	25–	1.95 (1.55, 2.47)	2.86 (2.15, 3.82)	5.44 (3.66, 8.08)
	35–	2.97 (2.38, 3.70)	4.72 (3.59, 6.20)	10.61 (7.32, 15.37)
	45–	6.28 (5.01, 7.86)	11.19 (8.51, 14.72)	27.06 (18.7, 39.16)
	55–	9.30 (7.32, 11.82)	18.10 (13.59, 24.10)	43.77 (29.97, 63.94)
	65–79	12.49 (9.41, 16.59)	24.01 (17.35, 33.22)	55.71 (36.81, 84.3)
Education	Junior school	1.00	1.00	1.00
	Junior high school	0.60 (0.52, 0.71)	0.59 (0.50, 0.69)	0.58 (0.49, 0.69)
	High school	0.53 (0.45, 0.63)	0.54 (0.45, 0.64)	0.56 (0.46, 0.68)
	Undergraduate	0.37 (0.31, 0.43)	0.33 (0.28, 0.39)	0.32 (0.26, 0.38)
Family Income (Chinese Yuan)	<500	1.00	1.00	1.00
	500–	0.76 (0.62, 0.92)	0.75 (0.61, 0.93)	0.73 (0.58, 0.91)
	1000–	0.64 (0.53, 0.77)	0.62 (0.51, 0.75)	0.62 (0.50, 0.76)
	2000–	0.67 (0.55, 0.82)	0.66 (0.54, 0.81)	0.63 (0.51, 0.79)
	3000–	0.55 (0.43, 0.70)	0.54 (0.42, 0.69)	0.50 (0.38, 0.66)
Occupation	Other *	1.00	1.00	1.00
	Manual labor	0.90 (0.76, 1.05)	0.83 (0.70, 0.97)	0.76 (0.64, 0.90)
	Mental labor	0.56 (0.47, 0.68)	0.48 (0.40, 0.59)	0.47 (0.38, 0.57)

* “other” included unemployed and retired people; The adjusted ORs for gender were adjusted for age, the adjusted ORs for age were adjusted for gender, and the adjusted ORs for education, family income, and occupation were adjusted for gender and age.

## 4. Discussion 

High levels of CVD risk factors constitute a major challenge to public health, but they are very common in many developing countries, including China. Although a number of researchers have investigated CVD risk factor clustering in China, little is known about the prevalence and clustering of the CVD risk factors that are associated with demographics in Jilin, China. This is the first study to report the prevalence and clustering of the main CVD risk factors by demographics in Jilin Province. 

Table 6 shows the prevalence of CVD risk factors in previous studies in China, and the prevalences of diabetes and overweight in our study were much higher than those in other studies. Jilin Province is located in the central of northeast China, with a temperate continental monsoon climate and an annual average temperature of 4.8 °C [27]. This type of climate usually requires a special diet such as eating more animal fat, more salt and fewer fresh vegetables. Moreover, the population participates in fewer outdoor activities (exercises), especially during the cold winter months. Therefore, the geographical features and the characteristics of our study population might have contributed to the high prevalence of diabetes and overweight.

**Table 6:** The prevalence of CVD risk factors in previous studies (%).

Author	Hypertension	Diabetes	Dyslipidemia	Overweight	Smoking	Survey Time and Region
Our study	37.3	8.2	36.8	47.3	31.0	2012, Jilin
Gu *et al.* [16]	26.1	5.2	53.6	28.2	34.5	2000–2001, China
Zhang *et al.* [28]	36.6	6.5	35.4	36.2	36.3	2007, Beijing
Xu *et al.* [4]	62.4	6.4	42.7	34.3	6.1	2011, Tibetan

However, the prevalences of dyslipidemia and smoking decreased compared with previous values, especially smoking, which might have been caused by China’s previous anti–smoking propaganda during these years. This might also partly explain the associations between high education levels and family incomes with the low prevalences of CVD risk factors. The reason for this was that persons with more education and/or higher family incomes have greater possibilities to engage in healthy lifestyles, including smoking less.

The prevalence of hypertension was slightly higher compared with the findings from previous studies. On one hand, people now live longer than before with improved medical treatments, while the prevalence of hypertension among the elderly is relatively higher. On the other hand, with improved living conditions, people now eat more meat, which might also have led to higher prevalence of hypertension. 

In the present study, 51.6% of males and 9.1% of females were smokers, and this prevalence was significantly higher in males than in females. In addition, the prevalences of the other four CVD risk factors in males were also higher than in females. Therefore, our study revealed that gender might be associated with CVD risk factor clustering. On the contrary, the prevalences of all the five risk factors did not differ significantly by residence (*p* > 0.05), due to the continuously decreasing gap between town and rural in Jilin. The prevalences of hypertension and diabetes increased with age, but the peak prevalences of dyslipidemia and overweight appeared in the subjects aged 55~64 years. Concurrently, the prevalences of all risk factors differed significantly by education level, family income and occupation. 

Further, it was revealed that gender, age, education level, family income and occupation were associated with the clustering of CVD risk factors (*p* < 0.05) via the multivariate logistic regression. In general, the adjusted ORs of having ≥1, ≥2, and ≥3 major CVD risk factors for males were 3.70, 4.66, and 5.76 in our study and 3.4, 4.3 and 5.4 in the study by Zhang *et al.* [28], which was consistent with our study. However, the same adjusted ORs in Gu *et al.* [16] were 2.61, 3.55 and 4.97, which were extremely lower than those in our study. The higher ORs in our study might have been the result of the different study populations and survey times. In addition, age was a risk factor for CVD risk factor clustering, and the adjusted ORs of having ≥1, ≥2, and ≥3 major CVD risk factors increased progressively with age, which was consistent with previous studies [15,29]. However, education level and family income were protective factors for CVD risk factor clustering. Hence, investment in education should be a concern as well.

Some limitations of our study should be noted. One was that former smoking was not encompassed in our investigation, but it was clear that former smoking had implications for CVD development. The other was that the respondents’ smoking status was based on self-report, which may be subject to reporting bias. 

## 5. Conclusions

CVD risk factor clusters are common among adults in Jilin, China, and they constitute a major public health challenge. Clearly, more effective prevention efforts that target CVD risk factors are needed in males, the elderly, and persons with less education and low family incomes as well as manual laborers. In addition, more feasible population-based interventions, such as advocating for smoking cessation, healthy diet, and increased physical activities, are suggested to reduce the prevalence and the clustering of CVD risk factors. Finally, a systematic large-scale educational effort that is directed in particular toward residents is also needed.

## Supplementary Files

Supplementary File 1
