# Supplementary material for: Risk Factors for Cardiovascular Disease and Their Clustering among Adults in Jilin (China)
Source: Int J Environ Res Public Health. 2015 Dec 23;13(1):70. doi: 10.3390/ijerph13010070 (PMC4730461; doi:10.3390/ijerph13010070)
Supplement: Supplementary File 1 [file ijerph-13-00070-s001.pdf]

Risk Factors for Cardiovascular Disease and Their Clustering among Adults in Jilin (China)

Online Supplementary Material

1. Study Population

We carried out a large-scale community-based cross-sectional health interview and examination survey on permanent residents aged 18 to 79 years old who had lived in Jilin province for more than six months. The survey respondents were asked to answer each question face-to-face with investigators who had been uniformly trained.

2. Inclusion and Exclusion Criteria

Inclusion criteria: people aged 18 to 79 years old who had lived in Jilin province for more than six months. Exclusion criteria: people where any variable was missing (*i.e., gender, age, residence, education, family income occupation, height, weight, diastolic blood pressure, systolic blood pressure, total cholesterol, triglyceride low-density, lipoprotein cholesterol, high-density lipoprotein cholesterol, fasting blood glucose*).

3. Sampling Size

There were 27,462,297 residents in Jilin province, in which 82% people were 18 to 79 years old, according to the main data bulletin of the 6th national population census in 2010 (announced by Statistical Bureau of Jilin province, 6 May 2011). The expected sample size is 22,520, which was 1‰ of the whole population in Jilin province. However, the planned sample size is 23,050, in consideration of the follow-ups lost during the field investigation.

4. Sampling Method

Five-stage stratified random cluster sampling was used to select the samples under study. In the first stage, 32 districts/counties were identified in proportion to population, geographic location and ethnicity, from nine cities (Changchun, Jilin, Siping, Liaoyuan, Tonghua, Baishan, Songyuan, Baicheng and Yanbian). At the second stage, three or four towns (depending on the size of the district) were selected by stratified random sampling to guarantee the representativeness of each sample. In the third stage, three neighborhood committees were chosen by stratified random sampling from each of the towns previously selected. In the fourth stage, one village from each chosen neighborhood committee was selected by simple random sampling. In the final stage, cluster random sampling was used to identify individuals aged 18 to 79 years old from each of the villages selected for the study.

5. Data Collection

A strict quality control system was implemented at each stage of the data collection to ensure uniformity and accuracy of the data. Before the formal investigation, a pre-investigation was conducted to explore the design of the questionnaire. In addition, systematic training for all investigators was organized to teach them on how to administer the screening questionnaire, and how to take anthropometric measurements. Before the interview, the identity of each participant was confirmed by the investigator. The validity of each answered questionnaire was examined by the interviewer after the participant had completed the questionnaire in order to ascertain whether the responses were consistent with the real situation. After the fieldwork, all data were processed by parallel double entry. Three verifications were carried out to check for incomplete or inconsistent responses, and then deleted the missing data that cannot be repaired.

The data collection included an intensive investigation and household survey. The purpose of the investigation was explained to the participants before the interview. Then given the option to sign an informed consent form, the participants were proceeded to answer the questions and do the further investigations for those who consented to participate in the study. The study was approved by the Institutional Review Board of the School of Public Health, Jilin University, Jilin, China. The demographic information collected included gender, age, family income, level of education, occupation, smoking, drinking, exercise, diagnosis and treatment of diabetes, and self-reported family history of diabetes.

6. Data Measurement

Anthropometric measurements including height, weight, blood pressure, serum lipids and fasting blood sugar were taken. During the interview, weight and height were determined though standardized protocol and measured in light indoor clothing without shoes. Body mass index (BMI) was calculated as weight in kilograms divided by height in meters squared (kg/m^2).

A mercury sphygmomanometer was used to measure the blood pressure in the sitting position after a 10-min rest period. The appearance of the first sound was used to define systolic blood pressure (SBP) and the disappearance of sound was used to define diastolic blood pressure (DBP). Two readings each of SBP and DBP were recorded, and the average of each measurement was used for data analysis. If the first two measurements differed by more than 5 mmHg, additional readings were taken.

Blood samples were obtained from the antecubital vein into anticoagulant tubes containing EDTA in the morning after an overnight fasting period. All of the collected samples were transported on dry ice at prearranged intervals to the central laboratory. Serum lipids including TC, TG, HLD-C and LDL-C, which were measured by a MODULE P800 biochemical analyses machine.

Fasting plasma glucose (FPG) levels were measured using the Bayer Bai Ankang fingertip blood glucose monitor machine by taking a small drop of blood from a finger onto a strip of paper in the morning after participants fasted for 10 or more hours overnight.

© 2015 by the authors; licensee MDPI, Basel, Switzerland. This article is an open access article distributed under the terms and conditions of the Creative Commons Attribution license (http://creativecommons.org/licenses/by/4.0/).
